# Mortality of Children from First Dentition

**Published:** 1853-10

**Authors:** 


*Mortality of Children from first Dentition.*—In the April No. of the American Journal of Dental Science for 1853, there is a well written article on the above subject, by Dr. J. L. Levison, in which the following interesting case is given:

"During my residence at Hull, in Yorkshire, occasionally some professional engagements led me, from time to time, to visit Louth, a beautiful town in Lincolnshire, the inhabitants of which were, many of them, independent people, so that during my brief sojourn I had always plenty of occupation—particularly as my practice embraced many of the surrounding towns. It was on one of my visits, that Mrs. S—— called to ask me to see her little nephew, whom she described to be about one-and-twenty months old—that he had not a single tooth through his gums, that he had most awful fits, and that with all their care and watchfulness they could not prevent them; that they came on at all times, occasionally remaining for hours, and that during the fit his body was stiff, resembling one already dead. That it was frightful to see him, as his eyes seemed as if they would start from their sockets, that he had not any power to move them, and that when so conditioned, he had not any volition over the sphincters of the rectum or bladder, and she concluded by saying—'will you see him, for he is altogether a most awful looking being?'

"Having ascertained that a medical man was attending him, and that her relatives resided eight miles in the country, I declined doing so, but from my personal friendship for the lady, I said '*his gums ought to be lanced.*' They have been, was her reply. Not sufficiently so, I remarked, or else the symptoms would be mitigated. That same day she drove into the country and reported our conversation to her relatives. The next morning while at breakfast a carriage stopped at my lodgings, and to my surprise Mrs. S——, accompanied by a servant carrying her nephew, were ushered into my room; and whilst the lady was apologising for, in a measure of forcing me to see the child, a fit came on, which was indeed most frightful. The *tetanic* state of the muscles—the eyes thrust forward without either motion or expression—the involuntary action of the rectum and bladder—the deadly bluish hue of the skin, made altogether such a painful spectacle, which with the urgent entreaties of the aunt to do something, that however desirous of obeying professional usages, my humanity could not resist the appeal—so taking the boy, I said, 'my dear madam as soon as the lancet touches the first tooth the fit will cease, and though it is my intention to cut down on every other tooth on both the jaws, the young gentleman may scream most lustily, (for he was a powerful made child,) but he will never have any repetition of the convulsions or the loss of consciousness.' The result was as I had predicted. And the fit, which had hitherto lasted for hours, was banished as if by magic in a few seconds by the potency of a good incision, whose mandate was distinctly expressed as the lancet grated on the tooth."

